# Region-Specific Reductions in Morphometric Properties and Synaptic Colocalization of Astrocytes Following Cocaine Self-Administration and Extinction

**DOI:** 10.3389/fncel.2018.00246

**Published:** 2018-08-07

**Authors:** Anze Testen, Marian T. Sepulveda-Orengo, Christiann H. Gaines, Kathryn J. Reissner

**Affiliations:** ^1^Curriculum in Neuroscience, University of North Carolina at Chapel Hill, Chapel Hill, NC, United States; ^2^Department of Psychology and Neuroscience, University of North Carolina at Chapel Hill, Chapel Hill, NC, United States

**Keywords:** cocaine, astrocyte, self-administration, nucleus accumbens, amygdala, prefrontal cortex, Lck-GFP

## Abstract

While much is known about the effects of cocaine use on the cellular structure and function of neurons and synapses within the brain’s reward circuitry, relatively little is known about the effects of cocaine on astrocytes. Given the significant role that astrocytes play in modulating neuronal and synaptic function, this lack of knowledge regarding the role of astroglial adaptations in the neuropathology of drug abuse represents an important investigative need. We recently showed that astrocytes within the nucleus accumbens (NAc) core exhibit decreased volume, surface area, and synaptic colocalization following cocaine self-administration and extinction, compared to NAc astrocytes from saline-administering animals (Scofield et al., 2016b). However, it is unknown whether these cocaine-dependent changes in astrocytes are ubiquitous throughout the brain’s reward circuitry, or represent specific adaptations within the NAc. It is also not known whether the extinction period is necessary for the retracted phenotype, or whether self-administration alone is sufficient to drive these changes. In the current study, we have extended our assessment of the effects of cocaine self-administration on morphometric properties and synaptic colocalization of astrocyte peripheral processes in the prelimbic region of the medial prefrontal cortex (PL) and basolateral nucleus of the amygdala (BLA), both known to also contribute significantly to motivated behaviors. In addition, in order to pinpoint the temporal dimension of previously observed effects, we also examined astrocytes within the NAc following the last self-administration session. While a reduction of astrocyte size and synaptic colocalization was observed in the NAc core of cocaine-extinguished rats as previously shown, no differences in PL or BLA astrocytes were observed between saline- and cocaine-extinguished rats. Moreover, decreased synaptic colocalization of peripheral processes in the NAc was observed with a post-synaptic marker, instead of a presynaptic marker as used previously. In contrast, no significant changes were found in NAc astrocytes after self-administration alone. These results provide insights into the influence of cocaine use on astrocytes within the brain reward circuitry, and inform both regional heterogeneity as well as temporal dynamics of astrocyte responsiveness to cocaine self-administration.

## Introduction

The rodent self-administration model of drug abuse has allowed for investigation of cellular consequences of drug use, as well as the circuitry and mechanisms that mediate motivated drug-seeking behaviors (Venniro et al., 2016). For example, self-administration studies combined with pharmacological inactivation of brain nuclei and more recently optogenetics, have been instrumental in elucidating the neural circuitry engaged in different stages within the cycle of substance use, dependence, abstinence, and relapse (Saunders et al., 2015; Cooper et al., 2017). Further, genetic manipulation has revealed cell subtype specific roles in drug reward and motivation (Lenz and Lobo, 2013), and self-administration studies have provided valuable insight into the subcellular signaling pathways and receptor dynamics which mediate drug use, dependence, and seeking (Panlilio and Goldberg, 2007; O’Connor et al., 2011; Scofield et al., 2016a; Wolf, 2016).

While use of this preclinical model has revealed considerable insight into drug-induced adaptations among neurons, relatively less is known about the consequences of self-administration on properties of astrocytes (Scofield and Kalivas, 2014). Dysregulation of glutamate homeostasis, largely accomplished by astrocytes, was among the first astroglial adaptations observed in cocaine-withdrawn animals, a response that has now been reported across numerous drug classes (Scofield et al., 2016a). Specifically, impairments in glutamate homeostasis as a consequence of drug use and withdrawal are mediated by decreased expression and activity of the cystine-glutamate exchanger xC- and it’s catalytic submit xCT, as well as the high affinity glutamate transporters GLT-1 and GLAST (Baker et al., 2003; Kalivas, 2009; Knackstedt et al., 2010a; Reissner et al., 2011). Notably, both system xC-, GLT-1, and GLAST are predominantly localized on astrocytes (Perego et al., 2000; Danbolt, 2001; Huang et al., 2004; Roberts et al., 2014). The regulation of these systems has been recognized as critical to drug seeking behaviors, illustrating functionally relevant roles for drug-induced adaptations to astrocytes.

Recently we reported that cocaine self-administration and extinction results in a decrease in surface area, volume, and presynaptic colocalization of astrocyte peripheral processes in the NAc core, suggesting impaired function and synaptic modulation by astrocyte processes (Scofield et al., 2016b). The observation that peripheral processes of astrocytes colocalize significantly less with synaptic puncta in the NAc core raises important hypotheses regarding astroglial modulation of synaptic function in drug-withdrawn animals, and by extension, drug-seeking behavior. While the NAc is an important node within the reward circuitry, and is considered a limbic-motor integrator (Mogenson et al., 1980), it is entirely unknown whether the observed phenomenon is restricted to the NAc core, or extends to other nuclei within the brain’s reward circuitry. Toward that end, in the current study we assessed astrocyte morphometric properties and synaptic colocalization of peripheral processes in the NAc core, BLA, and PL. To capture and measure morphometric changes in astrocytes, a membrane-tagged Lck-GFP reporter was expressed under the control of the GfaABC1D promoter as reported previously (Scofield et al., 2016b). Post-translational modification of a short amino acid sequence taken from the Lck and Fyn members of the Src family of protein tyrosine kinases leads to membrane association of GFP and subsequent enhanced visualization of fine peripheral astrocyte processes (PAPs) over soluble protein reporters (Zlatkine et al., 1997; Benediktsson et al., 2005; Shigetomi et al., 2013). The microinfusion of the viral vector was used to transduce the PL, NAc, and BLA of each rat. Animals were then trained in self-administration and extinction of cocaine or saline, and properties of astrocytes within these regions were subsequently assessed. We further assessed whether decreased synaptic colocalization of NAc peripheral processes would be observed using a post-synaptic marker, post-synaptic density protein 95 (PSD-95), in addition to a presynaptic marker, synapsin I, as reported previously (Scofield et al., 2016b), and whether changes are observed 24 h after the last self- administration session.

## Materials and Methods

### Laboratory Animal Care and Surgical Procedures

Adult male Sprague-Dawley rats (200–250 g at arrival) were obtained from ENVIGO (formerly Harlan Labs, Indianapolis, IN, United States) and housed individually in a temperature- and humidity-controlled, AAALAC-accredited environment on a reverse light-dark cycle (7 p.m. to 7 a.m. lights on). The reverse light cycle was used in this study and others (Scofield et al., 2016b) due to evidence that a reverse light-dark cycle can produce more stable behavioral responses in nocturnal rodents (Roberts et al., 2002). All handling, surgeries and behavior were performed during the dark cycle. During the 1 week of acclimatization and an additional week of post-surgery recuperation, animals had *ad libitum* access to food and water. All experiments and procedures were approved by the University of North Carolina at Chapel Hill Institutional Animal Care and Use Committee (IACUC) and were in accordance with NIH guidelines.

On the day of surgery, rats were anesthetized with ketamine (100 mg/kg) and xylazine (7 mg/kg) followed by a single dose of the analgesic ketorolac (0.3 mg/kg). A silastic catheter was surgically inserted into the right jugular vein, exiting from the back and ending with an exposed 22-gauge cannula (Plastics One, Roanoke, VA, United States). Catheters were flushed daily with the aminoglycoside antibiotic gentamicin (100 mg/ml, 0.1 ml) and heparin (100 U/ml, 0.1 ml) until the end of behavioral training. Immediately following catheterization, animals were stereotaxically microinjected with the lymphocyte protein kinase Lck-GFP reporter, packaged into the adeno-associated virus serotype 5 (AAV5), under the control of the GfaABC1D promotor (1.3 × 10^13^ virus particles/ml) (Shigetomi et al., 2013; Scofield et al., 2016b). Targeted areas were prelimbic cortex (PL, bilaterally; coordinates (mm): +3.2 anterior/posterior, +0.4 medial/lateral, -4.0 dorsal/ventral), basolateral amygdala (BLA, bilaterally; coordinates (mm): -2.8 anterior/posterior, +5.0 medial/lateral, -8.7 dorsal/ventral), and nucleus accumbens core (NAc, unilaterally; 6° angle, coordinates (mm): +1.5 anterior/posterior, +2.6 medial/lateral, -7.2 dorsal/ventral). Virus was microinjected using 26-gauge injection cannulas (Plastics One, Roanoke, VA, United States) in a single and dual configuration (1 μl per injection site, 0.05 μl/min injection rate) and left to diffuse for 15 min. Bilateral injections were made to PL and BLA, and unilateral injections to the NAc. After diffusion, microinjectors were slowly removed over 1–2 min.

### Behavioral Training

Catheterized and microinjected rats were randomly divided into two groups, self-administering either saline (*n* = 20, total across experiments) or cocaine (*n* = 24, total). Saline-administering rats were not yoked to cocaine-administering rats. Training took place in modular rat operant chambers (Med Associates, Latham, NY) at the same time each day. Cocaine self-administration was performed on a fixed-ratio 1 (FR1) schedule of reinforcement during 2 h sessions. An active lever press produced a 0.2 mg intravenous (i.v.) infusion of cocaine (or saline) as well as an auditory and visual cues, followed by a 20 s time-out period. Cocaine intake was capped for the first 3 days (maximum of 40 infusions) and unrestricted afterward. Threshold for minimum intake was 10 days of at least 10 cocaine infusions per day. When applicable, the self-administration phase was followed by 15 days of 2 h daily extinction sessions where cocaine, as well as contingent cues, were not available. Prior to the onset of behavioral training, rats received a single food training session (6 h), to facilitate acquisition of the operant task. Rats received 20 g of chow (45 g non-flavored pellets, BioServ, Flemington, NJ, United States) following each daily session throughout the study. Water was unrestricted. Cocaine was generously provided by the National Institute of Drug Abuse (NIDA) Drug Supply Program.

### Tissue Processing

Rats were deeply sedated with pentobarbital 24 h after the last extinction or self-administration session, followed by transcardial perfusion using 200 ml of phosphate buffer (1× PB) and 200 ml of freshly made 4% paraformaldehyde (in 1× PB). The time point of 24 h after the last extinction session was chosen, so that adaptations in astrocytes present at the time of a typical reinstatement test could be observed. Korogod et al. (2015) compared cryo and chemical fixation of the mouse neocortex and concluded that chemical fixation, even though inferior for answering some anatomical questions, reveals better preservation of glial volume and more intimate glial coverage of the synapses. Brains were then extracted, post-fixed for 3 h in 4% paraformaldehyde (4°C), washed by excess amount of cold 1× PB and stored for 2 days in 30% sucrose solution (in 1× PB) at 4°C. Tissue was sectioned (100 μm) using a cryostat (Leica Biosystems Inc., Buffalo Grove, IL, United States) and stored at -20°C in 50/50 Glycerol/PB until staining. The complete areas of NAc, PL and BLA were collected.

Before staining, each selected section was inspected for sufficient expression and anatomical targeting. Immunohistochemistry was performed on free-floating sections. Sections were washed (3 min × 5 min) in 1× PBS to remove any residual glycerol and blocked for 1 h at room temperature in 5% normal goat serum (NGS, Sigma-Aldrich, St. Louis, MO, United States) containing 1% Triton X-100 (Thermo Fisher Scientific, Waltham, MA, United States) in 1× PBS (1% PBST). Blocking solution was replaced with primary antibodies (all 1:500) in 5% NGS in 0.4% PBST: Rb anti-GFAP (Z0334, Dako) and Ms anti-PSD-95 (6G6-1C9, Thermo Fisher Scientific, Waltham, MA, United States). Sections were incubated for 72 h at 4°C with constant shaking, and were turned over half way through.

To probe with secondary antibodies, sections were transferred to new 24-well plates and washed with cold 1× PBS (3 min × 10 min). Secondary antibodies (goat anti-Rb Alexa Fluor 647 (A32733, Thermo Fisher Scientific, Waltham, MA, United States) and goat anti-Ms Alexa Fluor 594 (A11032, Thermo Fisher Scientific, Waltham, MA, United States), all 1:1,000) were added to 5% NGS in 0.4% PBST and incubated 72 h at 4°C identically as for primaries. Sections were then once again washed three times with 1× PBS, mounted and immediately cover-slipped (Dapi-fluoromount-G, SouthernBiotech) to prevent desiccation.

### Data Acquisition and Processing

**FIGURE 1:**
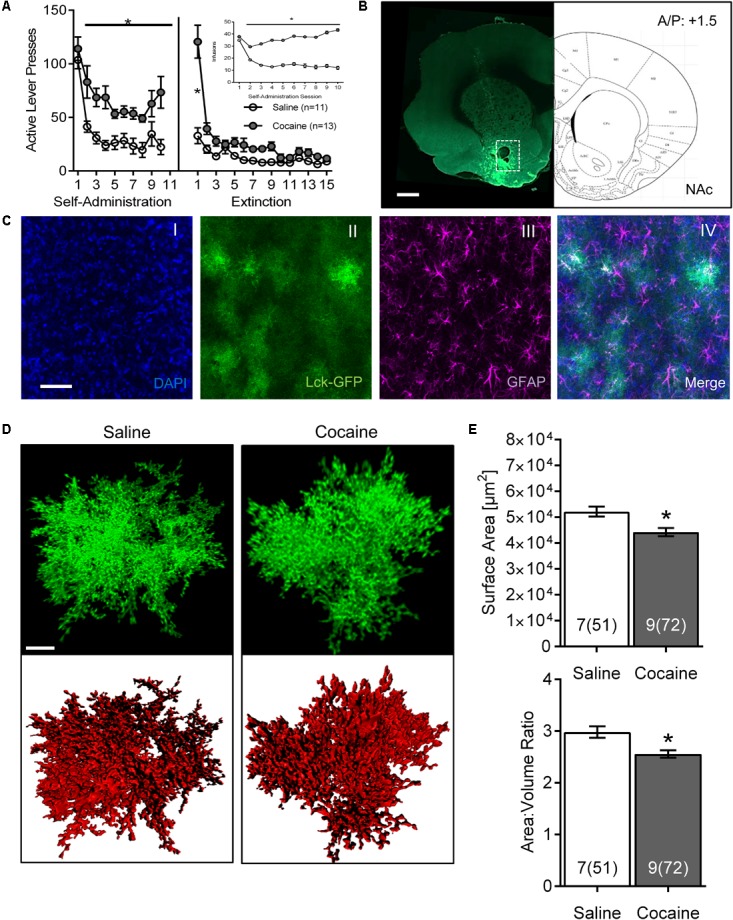
Cocaine self-administration and extinction reduces astrocyte surface area and surface area:volume ratio in the NAc core. **(A)** Active lever presses by session across self-administration (10 days) and extinction (15 days) training. Insert: number of infusions during self-administration sessions (^∗^*p* < 0.05 by two-way ANOVA). **(B)** Scan of a brain slice showing the intensity and the range of expression of the AAV5 GfaABC1D-Lck-GFP. The white dotted box shows the area of NAc from which astrocytes were selected for imaging. 10× magnification, scale bar: 1 mm. **(C)** Multiple intensity projection (MIP) of an area within the NAc showing Lck-GFP expressing astrocytes (II) as well as staining for DAPI (I), GFAP (III), and merged image (IV). Colocalization between GFAP and Lck-GFAP is shown in white. 20× magnification, scale bar: 50 μm. **(D)** Representative isolated astrocytes from saline and cocaine-experienced rats imaged in the NAc. Lck-GFAP signal is shown in green and surface rendering used for volumetric measurements in red. 63× magnification, scale bar: 10 μm. **(E)** Astrocyte surface area and surface area:volume ratio in NAc are significantly decreased in cocaine-administering rats compared to saline-administering rats. Bar insert: number of animals and number of cells (in parentheses). ^∗^*p* < 0.05 by nested ANOVA.

**FIGURE 2:**
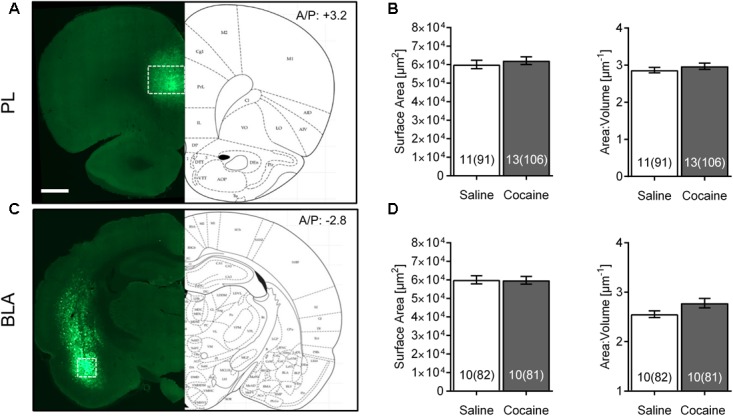
Cocaine self-administration and extinction does not affect morphometric features of PL or BLA astrocytes. **(A)** Scan of a brain slice showing the intensity and range of expression of the AAV5 Lck-GFP. The white box shows the area of PL where imaging was performed. 10× magnification, scale bar: 1 mm. **(B)** No significant difference was observed in astrocyte surface area and surface area:volume ratio in the PL following cocaine self-administration and extinction. Bar insert: number of animals and number of cells (in parentheses). **(C)** Lck-GFP expression in BLA. The white box shows the area where imaging was performed. **(D)** No significant difference was observed in astrocyte surface area and surface area:volume ratio in the BLA following cocaine self-administration and extinction. Bar insert: number of animals and number of cells (in parentheses).

To analyze astrocytes, optical sections of Lck-GFP-expressing cells were collected using a Zeiss LSM 800 confocal-scanning microscope (405, 488, 561, and 640 nm diode lasers; 2 Gallium Arsenide Phosphide (GaAsP) detectors) with a 63× oil-immersed objective (Zeiss, Oberkochen, Germany) and ZEN software suite (ZEN 2.3 (blue edition), Zeiss). Frame size was set to 1,024 × 1,024 pixels, bit depths to 16-bit, averaging to 4×, and z-step size to 1 μm. The imaging was restricted to previously mentioned regions (as marked in **Figures 1**, **2** with the white box) around A/P: 1.5, 3.2, and -2.8 for NAc, PL, and BLA, respectively. Astrocytes were imaged only if present in their entirety within the chosen region. Astrocytes bordering other regions or astrocytes being cut during sectioning in a z-dimension were not imaged. Cases in which viral injections missed their intended targets were not analyzed.

After acquisition, raw images were transferred to an image-processing workstation and deconvolved using AutoQuant software (v. X3.0.4, MediaCybernetics). A blind deconvolution algorithm with 10 iterations was run on each z-stack. The deconvolved output stack was directly imported to Imaris software (v. 8.4.1, Bitplane, Zürich, Switzerland) which was used to generate a 3-dimensional reconstruction of each individual astrocyte. Each individual cell was then isolated and a surface was built around it using Lck-GFP innate fluorescence signal. Surface area and volume were extracted from each individually built astrocyte surface. A special masked channel was generated using these surfaces, completely isolating the astrocyte Lck-GFP signal from Lck-GFP background. The masked channel was then used to perform colocalization analysis between the astrocyte Lck-GFP signal and the Alexa 594 signal, representing the PSD-95 post-synaptic neuronal marker. To remove the background, threshold for the PSD-95 channel was manually selected by taking repeated measurements of unambiguous puncta of fluorescent signal intensity on multiple optical slices. An average of these measurements was used as a final threshold for the PSD-95 channel. A new colocalization channel was generated which provided percentage of region of interest (ROI) colocalized with the PSD-95 channel (ROI was set as a masked Lck-GFP channel). It should be emphasized that fluorescence colocalization analysis is not a tool for detecting molecular interactions, but rather for assessing co-registry of two or more fluorophores within a given voxel. Accordingly, colocalization of Lck-GFP and PSD-95 signals serves as a proxy of astrocyte proximity to the neuronal post- synaptic terminal. The latter was checked by automatically counting PSD-95 puncta above a predetermined threshold within assigned 25 μm × 25 μm × 25 μm box for a subset of samples. Slides and files were encrypted during acquisition and manipulation to assure unbiased processing.

### Statistical Analysis

Collected data were grouped into tables using Excel 2016 (Microsoft, Redmond, WA, United States) and analyzed using Prism (v. 7.02, GraphPad, La Jolla, CA, United States) and SAS (v. 9.4, SAS Institute, Cary, NC, United States). Independent variables were saline versus cocaine self-administration and time, and dependent variables were lever presses and infusions (for behavior), and surface area, volume, and synaptic colocalization for astrocytes. Variance is reported as SEM. For analysis of the active lever presses and infusions during self-administration and extinction training, we used repeated measures two-way ANOVA. Nested ANOVA analysis was performed on all groups for assessment of morphometric properties, synaptic colocalization and PSD-95 expression (Heimer-McGinn et al., 2013; Risher et al., 2014). The comparison of properties of saline-treated astrocytes across brain areas (**Table 1**) was performed using Tukey-Kramer *post hoc* analysis, and comparison of behavior between cocaine and saline-administering rats on specific days (**Figure 1**) was performed using Bonferroni *post hoc* analysis. *p* < 0.05 was considered significant.

**Table 1:** Regional heterogeneity of GFAP-positive astrocytes.

	Surface area (μm^2^)	Volume (μm^3^)	Surface area: volume ratio (μm^-1^)
NAc	52,502 ± 3,114	19,259 ± 1,458	2.936 ± 0.114
PL	59,884 ± 2,441	22,016 ± 1,175	2.865 ± 0.090
BLA	59,225 ± 2,549	23,748 ± 1,220^∗^	2.570 ± 0.094^∗∗^

*Collected values from saline-extinguished rats from NAc, PL, and BLA. ^∗^Significantly different compared to NAc, ^∗∗^significantly different compared to NAc and PL. ^∗^, ^∗∗^ p < 0.05.*

## Results

### Cocaine Self-Administration and Extinction Training Leads to a Region-Specific Reduction in Astroglial Morphometric Features

Both active lever presses and cocaine infusions demonstrated main effects of group (cocaine versus saline) and time, and were significantly greater in cocaine-administering group by the 2nd day of SA, remaining elevated through self-administration [**Figure 1A**; infusions main effect of group *F*_(1,22)_ = 43.44, *p* < 0.0001 and time *F*_(9,198)_ = 8.74, *p* < 0.0001; self-administration active lever presses main effect of group *F*_(1,22)_ = 66.88, *p* < 0.0001 and time *F*_(9,220)_ = 11.48, *p* < 0.0001]. Main effects of group [*F*_(1,22)_ = 15.96, *p* < 0.001] and time [*F*_(14,308)_ = 30.82, *p* < 0.0001] were also observed across extinction, although *post hoc* differences between groups were only observed on extinction day 1.

The Lck-GFP protein exhibited astrocyte-specific expression, and the virus spread enabled single cell imaging of isolated, spatially labeled astrocytes (**Figures 1B**, **2A,C**). Slices were probed and imaged for the astrocytic marker GFAP, confirming that each imaged cell was indeed an astrocyte (**Figure 1C**). Cells were imaged only within targeted brain regions, as indicated by the white box in **Figures 1B**, **2A,C**. Regions that were not transduced, failed to show adequate spread, or were not within the ROI were excluded from analysis.

After acquisition, astrocytes were reconstructed in 3D space and surfaces were built using the Lck-GFP signal in Bitplane Imaris (**Figure 1D** and **Supplementary Video 1**). The sequential analysis revealed that the astrocytes in the NAc of cocaine-extinguished rats exhibited significantly decreased surface area [saline 52,155 ± 1,935 μm^2^, cocaine 44,252 ± 1,605 μm^2^, *F*_(1,14)_ = 6.93, *p* < 0.05] and surface-to-volume ratio [saline 2.983 ± 0.109 μm^-1^, cocaine 2.557 ± 0.072 μm^-1^, *F*_(1,14)_ = 4.79, *p* < 0.05] compared to astrocytes from saline-experienced rats (**Figure 1E**). Surface area:volume ratio was assessed as a proxy for astrocyte complexity, since the branching of peripheral processes contributes more to the surface area than the volume of the cell (Hama et al., 2004). Reduction in the number or length of these processes would thus report a lower ratio. There was no significant change observed in volume [saline 18,899 ± 1,347 μm^3^, cocaine 18,480 ± 1,137 μm^3^, *F*_(1,14)_ = 0.06, *p* > 0.05; **Supplementary Figure S1**].

Interestingly, none of these effects were observed in PL or BLA of the same rats (**Figures 2B,D**). No differences between astrocytes from the same saline- versus cocaine-extinguished rats were observed in surface area [PL saline 60,130 ± 2,270 μm^2^, cocaine 62,175 ± 2,084 μm^2^, *F*_(1,22)_ = 0.45, *p* > 0.05; BLA saline 60,036 ± 2,203 μm^2^, cocaine 59,787 ± 2,066 μm^2^, *F*_(1,18)_ = 0.01, *p* > 0.05], volume [PL saline 22,084 ± 976 μm^3^, cocaine 22,559 ± 953 μm^3^, *F*_(1,22)_ = 0.16, *p* > 0.05; BLA saline 24,240 ± 950 μm^3^, cocaine 23,173 ± 967 μm^3^, *F*_(1,18)_ = 0.54, *p* > 0.05] or surface area:volume ratio [PL saline 2.869 ± 0.073 μm^-1^, cocaine 2.970 ± 0.085 μm^-1^, *F*_(1,22)_ = 0.46, *p* > 0.05; BLA saline 2.555 ± 0.067 μm^-1^, cocaine 2.779 ± 0.095 μm^-1^, *F*_(1,18)_ = 2.45, *p* > 0.05].

We also probed for heterogeneity of astrocytes by comparing morphometric features of control animals across different brain regions (**Table 1**). Surface area:volume ratio of BLA astrocytes was significantly smaller compared to the NAc and PL (due to its significantly larger volume).

### Cocaine Self-Administration and Extinction Training Leads to a Region-Specific Decrease in Colocalization Between NAc Astrocyte Processes and the Neuronal Post-synaptic Marker PSD-95

**FIGURE 3:**
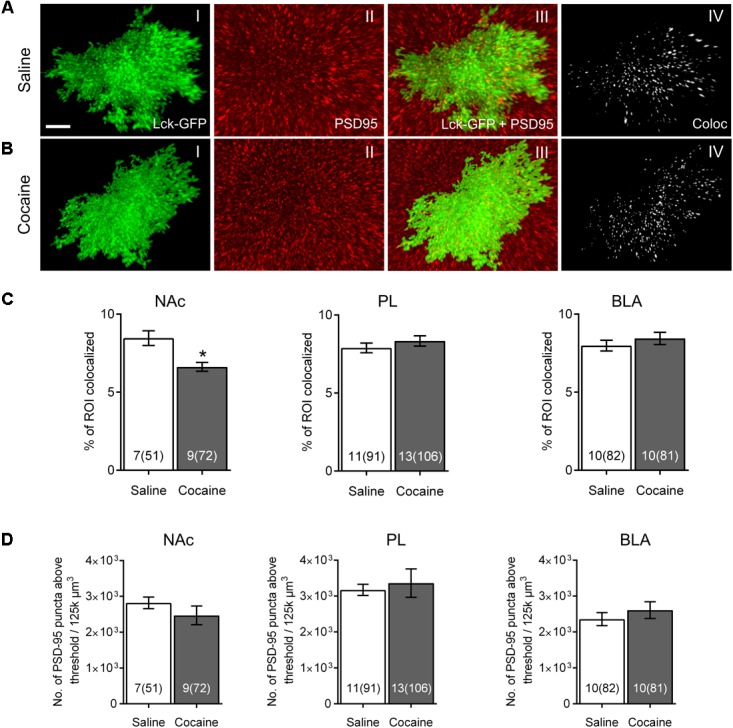
Colocalization between astrocytes and post-synaptic marker PSD-95 following cocaine SA and extinction in NAc, PL, and BLA. **(A)** Isolated Lck-GFP signal from a single NAc astrocyte (I) from a saline-experienced rat and the same area, stained for neuronal post-synaptic marker PSD-95 (II). Both signals were overlapped (III) and Imaris was used to isolate the PSD-95 signal colocalized with Lck-GFP (IV). **(B)** Same as in **(A)**, an astrocyte from a cocaine-extinguished rat. **(C)** Imaris was used to evaluate the percentage of isolated astrocyte Lck-GFP signal (ROI) colocalized with the PSD-95 signal. Significant difference in % of ROI colocalized between cocaine- and saline-administering rats was found in the NAc, but not in the PL or BLA regions. Bar insert: number of animals and number of cells (in parentheses). **(D)** Automated counting of PSD-95 positive puncta above the previously determined threshold level showed no difference in PSD-95 expression in any of the selected brain regions following cocaine versus self-administration and extinction. Bar insert: number of animals and number of cells (in parentheses). ^∗^*p* < 0.05 by nested ANOVA.

Previously we reported that colocalization of NAc astrocyte peripheral processes with a presynaptic marker, synapsin I, was significantly decreased in cocaine-extinguished rats (Scofield et al., 2016b). In the current study, we sought to determine whether this finding could be reproduced using a post-synaptic marker, PSD-95 (**Figures 3A,B**, **Supplementary Video 2**). We found that cocaine SA and extinction training resulted in a significant decrease in colocalization between GFP- and PSD-95-positive voxels in the NAc [saline 8.464 ± 0.469%, cocaine 6.623 ± 0.282%, *F*_(1,14)_ = 4.84, *p* < 0.05], but not in the PL [saline 7.892 ± 0.309%, cocaine 8.339 ± 0.336%, *F*_(1,22)_ = 0.67, *p* > 0.05] or BLA [saline 7.984 ± 0.344%, cocaine 8.443 ± 0.398%, *F*_(1,__18)_ = 0.31, *p* > 0.05] of the same rats (**Figure 3C**).

Importantly, one possible reason that a change in colocalization could be observed is if there is a difference in expression of either GFP or PSD-95 between groups. The percentage of colocalization when compared across brain regions in control animals was almost identical (colocalization not dependent on a region) as was the expression of PSD-95 as assessed by number of PSD-95-postive puncta above threshold [**Figure 3D**; NAc saline 2,819 ± 162, cocaine 2,469 ± 261, *F*_(1,14)_ = 0.72, *p* > 0.05; PL saline 3,172 ± 157, cocaine 3,361 ± 393, *F*_(1,__22)_ = 0.06, *p* > 0.05; BLA saline 2,359 ± 181, cocaine 2,610 ± 233, *F*_(1,18)_ = 0.47, *p* > 0.05]. We previously reported that cocaine self-administration and extinction had no effect of GFP-positive or synapsin I-positive puncta above threshold in the NAc core (Scofield et al., 2016b); accordingly, the decrease in post-synaptic colocalization of astrocytes is not likely due to a change in expression of either colocalized components.

### Extinction Training Is Necessary for Decreased Area, Surface-to-Volume Ratio and Neuronal Colocalization of NAc Astrocytes Following Cocaine Self-Administration

**FIGURE 4:**
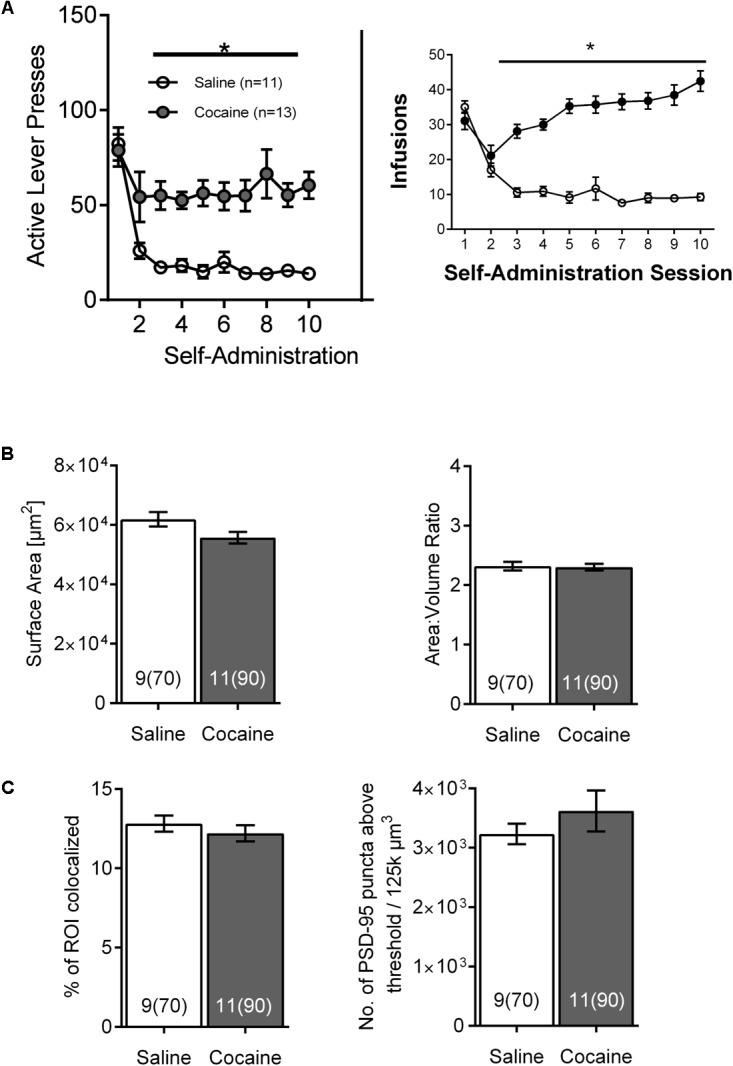
The effects of cocaine self-administration on NAc astrocytes is not observed 24 h following the last self-administration session. **(A)** Active lever presses and cocaine infusions by session across self-administration (10 days) (^∗^*p* < 0.05 by two-way ANOVA). **(B)** No significant differences were observed in surface area (left) or surface area:volume ratio (right) in NAc astrocytes following cocaine versus saline self-administration. Bar insert: number of animals and number of cells (in parentheses). **(C)** No significant differences were observed following self-administration in NAc astrocyte:PSD-95 colocalization (left) or in the PSD-95 expression (right). Bar insert: number of animals and number of cells (in parentheses).

To distinguish the temporal dynamics of the effects of cocaine self-administration on NAc astrocytes, a subsequent cohort of rats was sacrificed 24 h after the last SA session. Again, both active lever presses and cocaine infusions demonstrated main effects of group (cocaine versus saline) and time, and were significantly greater in cocaine-administering group by the 2nd day of SA, remaining elevated through self-administration [**Figure 4A**; infusions main effect of group *F*_(1,__18)_ = 69.9, *p* < 0.0001 and time *F*_(9,162)_ = 14.24, *p* < 0.0001; self-administration active lever presses main effect of group *F*_(1,__18)_ = 19.69, *p* < 0.0001 and time *F*_(9,162)_ = 13.10, *p* < 0.0001]. In this case, no significant changes were observed regarding astrocyte surface area [**Figure 4B**-left; saline 61,721 ± 2,770, cocaine 55,555 ± 2,447, *F*_(1,__18)_ = 2.78, *p* > 0.05] and surface-to-volume ratio [**Figure 4B**-right; saline 2.305 ± 0.09 μm^-1^, cocaine 2.317 ± 0.08 μm^-1^, *F*_(1,18)_ = 0.01, *p* > 0.05] as well as PSD-95 colocalization [**Figure 4C**-left; saline 12.754 ± 0.640, cocaine 12.212 ± 0.565, *F*_(1,__18)_ = 0.40] and PSD-95 expression profile [**Figure 4C**-right, saline 3,236 ± 307, cocaine 3,621 ± 278, *F*_(1,__18)_ = 0.86, *p* > 0.05].

## Discussion

### Cocaine Self-Administration and Extinction Leads to Reduced Morphometric Features and Synaptic Colocalization of NAc Astrocytes

Results provided herein reproduce and extend upon previously published findings regarding the effects of cocaine self-administration and extinction on NAc astrocytes (Scofield et al., 2016b), and further indicate that the reduced synaptic colocalization in the NAc can be observed using both pre- and post-synaptic markers. These results also indicate that the effects of cocaine self-administration and extinction are specific to astrocytes in the NAc, and require a period of extinction or extended withdrawal. Previous studies have also found decreased astroglial protein expression specifically in the NAc by Western blot, including GFAP, xCT, GLAST, and GLT-1 (Knackstedt et al., 2010a; Reissner et al., 2011; Scofield and Kalivas, 2014; Scofield et al., 2016b), supporting our observation that the effects of cocaine self-administration and extinction on astrocytes may be specific to this node of the reward circuitry. It is possible that the lack of change in BLA and PL astrocytes after extinction training might have been the result of the recovery of the normal phenotype during the extinction period. Nonetheless, at this time period following extinction, the observed effect is specific to NAc astrocytes over PL or BLA astrocytes. Accordingly, these results raise intriguing questions about both the mechanism and functional role of NAc-specific adaptations. What is unique about astrocytes in the NAc? The answer may lie in regional differences among astrocytes, or in the cellular environment of the NAc across the progression of cocaine self-administration and extinction, or both.

Regarding regional differences among astrocytes, elaborate astroglial morphology prompted early interest in diversity and heterogeneity of astrocytes (Prochiantz and Mallat, 1988; Wilkin et al., 1990; McKhann et al., 1997), yet these early findings were slow to be reexamined as compared to advances made within neuronal regional heterogenetity (Zhang and Barres, 2010; Khakh and Sofroniew, 2015). However, recent studies show that astrocytes integrated within different circuits exhibit markedly different morphological, proteomic, transcriptomic and functional profiles (Ben Haim and Rowitch, 2017; Chai et al., 2017), although it remains a challenge to ascribe functional differences among astrocytes within different nuclei or circuits. Moreover, even within specific nuclei, astrocyte populations with different molecular markers and different cell fates can be observed (Schitine et al., 2015; John Lin et al., 2017). Our results indicate morphometric heterogeneity of astrocyte responsiveness within the reward circuitry that will help to isolate specific astroglial contributions to maladaptive reward processing following drug abuse.

Regarding the cellular environment of the NAc, acute exposure to many drugs, particularly psychostimulants, leads to robust hyperdopaminergia within the NAc, which over time results in adaptations in glutamatergic and GABAergic signaling that may well contribute to the region-specific effects observed here (Mameli and Luscher, 2011; Luscher, 2016). Over time and repeated exposures, series of adaptations are observed, including plasticity of glutamatergic synapses (Uys and Reissner, 2011). In addition, some studies indicate cellular effects which occur across protracted periods of withdrawal. For example, Fischer-Smith et al. (2012) have observed an increase in the magnitude of GLT-1 protein suppression as a function of both cocaine use and withdrawal period. Accordingly, in order to determine whether the effects of cocaine self-administration on NAc astrocytes is a function of the pharmacological effects of cocaine, or is a function of withdrawal, we used a separate cohort of animals, to evaluate the effects of cocaine self-administration on morphometric properties and synaptic colocalization of NAc astrocytes. Indeed, no differences were observed between astrocytes from saline versus cocaine-experienced rats, supporting other studies which indicate that dynamic changes occur during a period of withdrawal from chronic self-administration. We cannot say conclusively whether the observed changes in NAc astrocytes were a function of extended withdrawal or of extinction training itself. It is well established that extinction training modulates synaptic plasticity in the NAc, augmenting changes in glutaminergic receptor expression and function (Self et al., 2004; Boudreau and Wolf, 2005; Knackstedt et al., 2010b; Wolf and Ferrario, 2010; Lee and Dong, 2011; Loweth et al., 2014; Scofield et al., 2016a). However, given that progressive reductions in GLT-1 are observed across protracted abstinence (Fischer-Smith et al., 2012; Kim et al., 2018), we believe that it is most likely that the observed changes may be a function of abstinence over extinction.

A large body of evidence indicates that astrocytes play important roles in the modulation of synaptic and neuronal function, including synaptic strength, and neuronal excitability (Barker and Ullian, 2010; Allen, 2014; Shibasaki et al., 2014; De Pitta et al., 2016; Singh and Abraham, 2017). Synaptic strength of glutamatergic projections onto NAc core medium spiny neurons is potentiated following withdrawal from chronic cocaine self-administration, and induction of long-term potentiation (LTP) and long-term depression (LTD) is impaired (Martin et al., 2006; Moussawi et al., 2009; Chen et al., 2010). Further studies will be critical to test hypotheses regarding the functional consequences of decreased synaptic colocalization on synaptic and neuronal function and plasticity.

### Limitations of the Study

While reduced surface area and surface area:volume ratios were observed in NAc astrocytes from cocaine-extinguished rats, we did not, however, observe the previously reported effect of cocaine self-administration and extinction on the overall volume of NAc astrocytes. One possible explanation for this observation could arise from the minor differences in tissue preparation and optical parameters leading to different baseline measurements. Notably, baseline surface area measurements of NAc astrocytes in saline-experienced rats were higher in the current study (approximately 5.0 × 10^4^ μm^2^; **Figure 1**) compared to approximately 2.7 × 10^4^ μm^2^ in the previous study (Scofield et al., 2016b), while baseline volume measurements were noticeably lower in the current study (1.5 × 10^4^ μm^3^, data not shown) compared to approximately 3.1 × 10^3^ μm^3^ in the previous study (Scofield et al., 2016b). In particular, the lower baseline volume measurements in the current study could well mask decreases in volume of astrocytes from cocaine-extinguished rats. Also in the current study, slide-mounted slices were coverslipped while still wet to minimize potential morphological changes introduced by a drying process; in contrast, tissue sections were dried prior to slide mounting in the previously reported study. Measurement differences could also be attributed to differences in imaging parameters on different microscopes between the two studies (Zeiss LSM 800 compared to Leica SP5 confocal microscopes). In the current study, the increased sensitivity of the detector (GaAsP), improved light source (diode laser versus argon laser in a previous study), as well as smaller z-stack acquisition step (1 μm compared to 2 μm in the previous study) all enabled more precise reconstruction, which could certainly influence baseline measurements. While overall results presented herein corroborate the previously reported decline in morphometric properties of NAc astrocytes in cocaine-extinguished rats, these findings also illustrate the importance of relatively minor experimental variables in the overall quantitative results, which will be critical to consider in further experimentation moving forward.

It is also important to note that colocalization is a function of the voxel size, determined by the microscopy system, and in this case, colocalization reflects distances between fluorophores within approximately 250 nm. While super resolution and electron microscopy indicate that astrocyte–synapse interactions can occur within tens of nanometers (Panatier et al., 2014; Heller and Rusakov, 2015, 2017; Heller et al., 2017), functionally relevant distances may well be higher. For example, astrocyte processes containing GLT-1 have been measured by immunogold electron microscopy at distances up to 400 nm from the active zone (Omrani et al., 2009). Further, a recent study using confocal colocalization analysis in the range of 10–600 nm observed changes in colocalization between peripheral processes and synapses following oxygen-glucose deprivation and in a genetic mouse model of Huntington’s Disease (Octeau et al., 2018). Moreover, changes reported here are observed for distances within 250 nm, including those much closer. Accordingly, decreases in colocalized signal include the most proximal interactions.

### Future Plans

Appreciation of the responsiveness of non-neuronal cells and neuroimmune signaling to drugs of abuse has escalated considerably in recent years (Clark et al., 2013; Cui et al., 2014). While it has been known for some time that drug abuse can influence blood-brain barrier integrity and immunity, only in more recent years has it become evident that non-neuronal cells may contribute to drug reinforcement, dependence, and mechanisms of relapse (Pellegrino and Bayer, 1998; Lacagnina et al., 2017; Nennig and Schank, 2017). While the current study is focused on investigation of the protracted effects of cocaine self-administration on astrocytes, we must consider this within the broader context of neuroimmune signaling within the brain. For example, microglia have been shown to become activated after non-contingent cocaine exposure (Little et al., 2009). Cocaine exposure is also associated with increased expression of nuclear factor kappa B (NF-κB), a central mediator of immune responsiveness (Ang et al., 2001; Russo et al., 2009) which initiates the cascade leading to a decrease in antioxidant defense and an increase in reactive oxygen species, which in turn further promote microglial NF-κB activation (Block and Hong, 2007). This and other neuroimmune pathways may well be engaged in the NAc by drug use, since it is known that activity of microglial NMDA receptors can activate NF-κB (Lipsky et al., 2001; Munhoz et al., 2008) leading to inflammation. Accordingly, continued investigation of both breadth and depth of non-neuronal and neuroimmune signaling following drug use will importantly expand on understanding of cellular mechanisms of drug use and addiction.

Within this context, results presented herein indicate a region-specific reduction of structure and synaptic colocalization of NAc astrocytes in cocaine-extinguished rats. This supports our previous report, as well as others indicating decreased expression and function of astroglial mediators of glutamate homeostasis following cocaine self-administration and extinction (Baker et al., 2003; Knackstedt et al., 2010a; Fischer-Smith et al., 2012; Scofield and Kalivas, 2014; Scofield et al., 2016b). However, it is important to note that other studies have reported evidence for reactive astrocytes in the NAc following non-contingent cocaine administration (Kim et al., 2017). For example, evidence for increased GFAP expression and reactive astrogliosis has been reported following non-contingent administration of cocaine, alcohol, methamphetamine, and opiates (Bowers and Kalivas, 2003; Udomuksorn et al., 2011; Friend and Keefe, 2013). Other studies have reported acute astrogliosis following a single non-contingent administration of cocaine, which is normalized following repeated administrations (Blanco-Calvo et al., 2014), as well as an ER stress-induced astrogliosis following a single 12 h *in vitro* cocaine incubation (Periyasamy et al., 2016). These differences illustrate important effects of pharmacodynamic responses to cocaine, and the importance influence of behavioral and administration approaches.

Relatively few studies have reported the effects of self-administered cocaine on properties of astrocytes. In addition to the report here and (Scofield et al., 2016b), a recent study found no effect on the number of GFAP-positive cells in the NAc core following a 12 h binge cocaine self-administration session, 14 days after the cessation of 10 days of cocaine self-administration (Wang et al., 2017). It remains to be determined how long-access cocaine self-administration or punishment-resistant self-administration affects astrocyte structure and physiology. Nonetheless, it is clear that there are measurable adaptations that occur in NAc astrocytes following cocaine exposure. Continued investigation moving forward will be critical to determine the functional consequences of these adaptations, and the role(s) that astrocytes play in the behavioral pathology of compulsive drug seeking and relapse to use.

## Ethics Statement

This study was carried out in accordance with the recommendations of the Guide for the Care and Use of Laboratory Animals, The Association for Assessment and Accreditation of Laboratory Animal Care International (AAALAC). The protocol was approved by the University of North Carolina at Chapel Hill Institutional Animal Care and Use Committee (IACUC).

## Author Contributions

AT and KR designed the study. AT, MS-O, and CG performed the surgeries and behavioral experiments. AT performed imaging and data analysis, and prepared the figures. AT and KR wrote the manuscript.

## Conflict of Interest Statement

The authors declare that the research was conducted in the absence of any commercial or financial relationships that could be construed as a potential conflict of interest.

## Supplementary Material

FIGURE S1 Volume of analyzed astrocytes did not change significantly in any of the studied brain regions following cocaine self-administration and extinction nor did it change after self-administration alone. Bar insert: number of animals and number of cells (in parenthesis).

VIDEO S1 Morphology and surface building around a single representative Lck-GFP expressing astrocyte in NAc.

VIDEO S2 Colocalization analysis of a single representative Lck-GFP expressing astrocyte and PSD-95 in NAc.
