# Supplementary material for: Region-Specific Reductions in Morphometric Properties and Synaptic Colocalization of Astrocytes Following Cocaine Self-Administration and Extinction
Source: Front Cell Neurosci. 2018 Aug 7;12:246. doi: 10.3389/fncel.2018.00246 (PMC6096402; doi:10.3389/fncel.2018.00246)
Supplement: FIGURE S1 — Volume of analyzed astrocytes did not change significantly in any of the studied brain regions following cocaine self-administration and extinction nor did it change after self-administration alone. Bar insert: number of animals and number of cells (in parenthesis). [file Image_1.pdf]

Figure S1

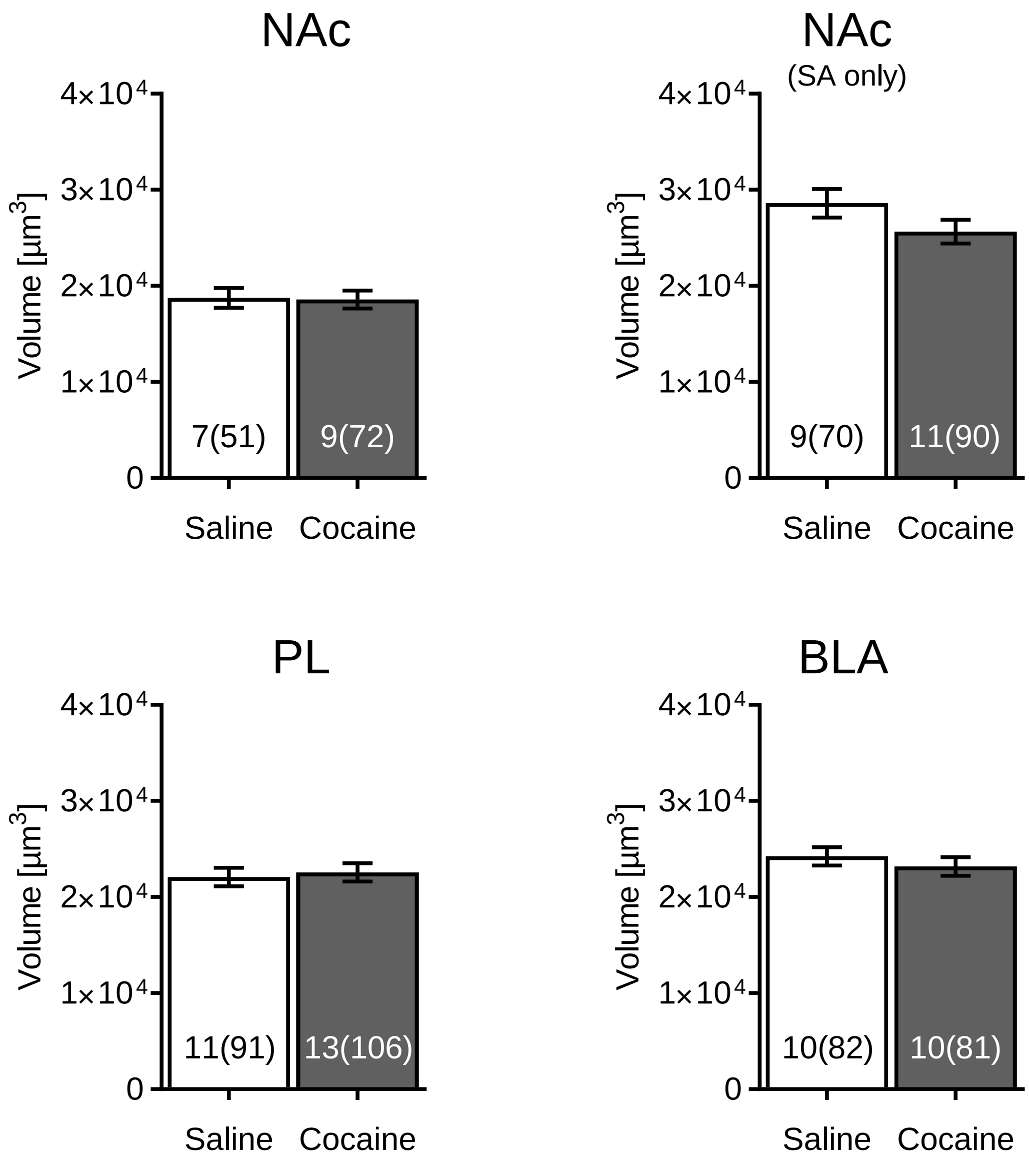

Figure S1: Volume of analyzed astrocytes did not change significantly in any of the studied brain regions following cocaine self-administration and extinction nor did it change after self-administration alone. Bar insert: number of animals and number of cells (in parenthesis).
